# Where Do Caregivers Take Their Sick Children for Care? An Analysis of Care Seeking and Equity in 24 USAID Priority Countries

**DOI:** 10.9745/GHSP-D-20-00115

**Published:** 2020-09-30

**Authors:** Sarah E.K. Bradley, Lauren Rosapep, Tess Shiras

**Affiliations:** a Abt Associates, International Development Division, Sustaining Health Outcomes through the Private Sector (SHOPS) Plus Project, Rockville, MD, USA.

## Abstract

Pneumonia, diarrhea, and malaria are leading causes of under-5 mortality. Accelerated reductions in illness burden are needed to meet childhood Sustainable Development Goals. Understand-ing where parents take sick children for care is key to improving equitable, high-quality treatment for these childhood illnesses and catalyzing reductions in morbidity and mortality. We analyzed the most recent Demographic and Health Survey data in 24 of the United States Agency for International Development’s maternal and child health priority countries to examine levels and sources of care for children sick with 3 illness classifications: symptoms of acute respiratory infection, diarrhea, or fever. On average, across countries analyzed, one-third of children had recent experience with at least 1 of the 3 classifications. The majority (68.2%) of caregivers sought external advice or treatment for their sick children, though the level is far higher for the wealthiest (74.3%) than poorest (63.1%) families. Among those who sought out-of-home care, 51.1% used public sources and 42.5% used private-sector sources. Although sources for sick child care varied substantially by region and country, they were consistent across the 3 illness classifications. Urban and wealthier families reported more use of private sources compared with rural and poorer families. Though 35.2% of the poorest families used private sources, most of these (57.2%) were retail outlets like pharmacies and shops, while most wealthier families who sought care in the private sector went to health facilities (62.4%). Efforts to strengthen the quality of integrated management of sick child care must therefore reach both public and private facilities as well as private pharmacies, shops, and other retail outlets. Stakeholders across sectors must collaborate to reach all population groups with high-quality child health services and reduce disparities in care-seeking behaviors. Such cross-sectoral efforts will build clinical and institutional capacity and more efficiently allocate resources, ultimately resulting in stronger, more resilient health systems.

## Abstract (precis)

Understanding whether and where parents take sick children for care is critical to improve child health and survival. Stakeholders should use this information to ensure that resources are programmed effectively and that sectors complement one another to increase equitable access to high quality integrated management approaches for sick child care.

## Teaser

**Key Findings:**

- Out-of-home care-seeking levels were similar for the 3 illness classifications, ranging from 63% for diarrhea to 70% for acute respiratory infection symptoms.
- Among caregivers who sought out-of-home treatment or advice for their sick children, 51% went to the public sector and 43% went to private sector sources.
- There are substantial disparities in illness prevalence and care seeking: 74% of caregivers from the wealthiest households and 63% of caregivers from the poorest households sought out-of-home care for their sick children.

**Key Implication:**

- Stewards of the public and private sectors—including governments, nongovernmental organizations, civil society, donors, and implementing partners must understand sources of sick child care and collaborate to improve access to and quality of affordable care.

## INTRODUCTION

In the last 30 years, there has been remarkable global progress in reducing child mortality. The under-5 mortality rate decreased by more than half, from 93 deaths per 1,000 live births in 1990 to 39 deaths per 1,000 live births in 2018.^1^ Yet, tremendous work remains. On average, 15,000 children died every single day in 2018.^1^

Progress in reducing child mortality has also been uneven: in an analysis across 137 low- and middle-income countries (LMICs), the under-5 mortality rate was more than 2 times higher among children from the poorest than the wealthiest families (65 versus 31 deaths per 1,000 live births, respectively).^2^ To meet global child health goals, the development community will need to focus on closing equity gaps and accelerating further reductions in child deaths, including those from pneumonia, diarrhea, and malaria. Together these 3 preventable and treatable illnesses account for nearly one-third of under-5 deaths.^1^^,^^3^

Many LMIC governments and donors are focusing on countries’ journeys to self-reliance to simultaneously transition away from donor dependence and increase gains in maternal and child survival.^3^^,^^4^ Key to this goal is collaboration and harmonization of efforts across all health sector actors. Global development stakeholders including the Gates Foundation, the United States Agency for International Development (USAID), United King-dom’s Department for International Development, and the Global Financing Facility have emphasized the importance of collaborating with governments, other donors, civil society, faith-based organizations, and the private sector effectively and efficiently to save women and children’s lives.^4^^–^^7^ USAID’s Private Sector Engagement Policy echoes and amplifies this message, calling on public and private sector actors to^8^:

*take the unique capabilities of each [sector] and apply them to problems that neither could address fully on their own*.

To facilitate such collaboration and harmonization, stakeholders first need to understand whether and where parents are seeking treatment for their sick children and how levels and sources vary across regions, countries, and population groups.

This study aims to provide updated information on levels and sources of care for children sick with symptoms of acute respiratory infection (ARI), diarrhea, and fever with an equity lens. We analyzed data from the most recent Demographic and Health Survey (DHS) data for 24 of the 25 USAID maternal and child health priority countries to address 3 research questions:

1. What is the prevalence of reported ARI symptoms, diarrhea, and/or fever among children under 5 in USAID priority countries?
2. How commonly do caregivers seek out-of-home care for their sick children?
3. When caregivers seek treatment or advice for their sick children, which sources do they use?

We aimed to provide the most up-to-date information on levels and sources of sick child care.

We examined equity implications for each research question and present notable differences in prevalence rates and care-seeking patterns by urban-rural residence and between households in the highest and lowest wealth quintiles of each country. We recognize that there is a great degree of overlap between socioeconomic status and urbanicity. For example, in 21 of the 24 priority countries analyzed, more than 90% of the poorest families live in rural areas. However, barriers related to these characteristics are different: care-seeking barriers related to geography are more likely to pertain to availability of and access to services whereas barriers related to socioeconomic status are more likely related to affordability. Therefore, we disaggregated findings by both residence and socioeconomic status to allow practitioners to understand differences in these populations and draw implications for programs and policies.

In the context of an increasing focus on private-sector engagement in health and using newly-available Demographic and Health Survey (DHS) data, we have built on previous analyses^9^^–^^11^ to provide the most up-to-date information on levels and sources of sick child care. We examined patterns across population segments with a focus on socioeconomic equity, noting the substantial progress that needs to be made. At the end of this paper, we describe and provide links to additional interactive data visualization resources and describe some of our work to date promoting the use of this information to transform research into action at the country level.

## DATA AND METHODS

### Data

**FIGURE 1.**
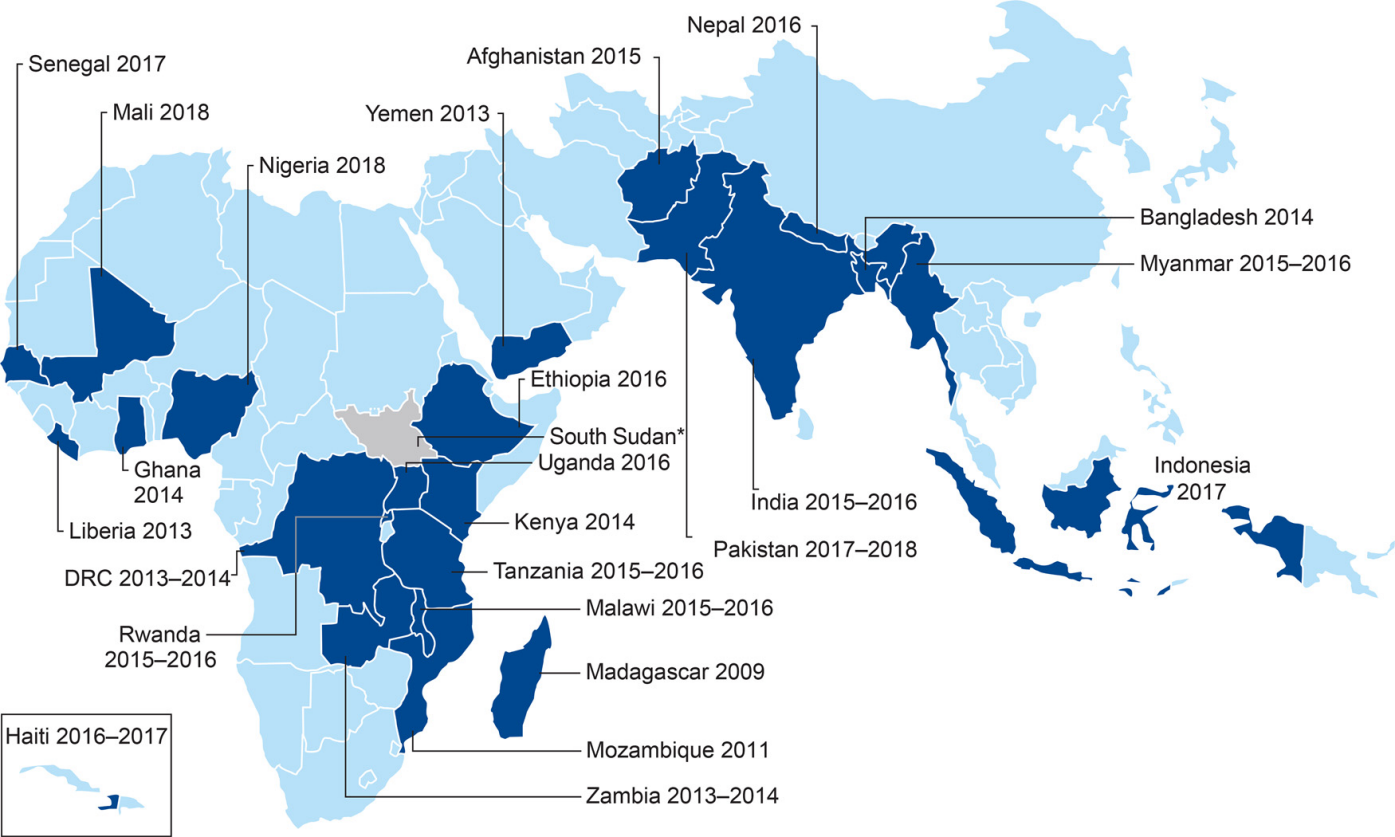
USAID Maternal and Child Health Priority Countries Analyzed Using Demographic and Health Survey Data^a^ Abbreviation: USAID, U.S. Agency for International Development.^a^ No Demographic and Health Survey data are available for South Sudan.

We analyzed the most recent nationally representative DHS household survey data from each priority country that was available on the DHS website as of December 31, 2019 (Figure 1). Data were available for all priority countries except South Sudan. Latest available data are quite recent for most countries except for Madagascar (from 2009), Mozambique (from 2011), and Yemen (from 2013). For these 3 countries, we note that data presented here may not accurately reflect the current situation.

#### Data on Prevalence of Illness Classifications

During DHS data collection, eligible women are interviewed and asked about each of their children. For each living child aged 5 years or younger, mothers are asked questions about whether their child had cough, diarrhea, or fever in the 2 weeks before the survey. If the child had a cough, the mother is asked whether the child had rapid or difficult breathing that was chest-related. Following DHS standards, we classify cough with rapid or difficult chest-related breathing as ARI symptoms, which are used as a non-specific proxy for pneumonia. Fever is used as a non-specific proxy for malaria. In this article, we refer to ARI symptoms, diarrhea, and fever as illness classifications, which are not confirmed by any diagnostic tests, but come directly from mothers’ reports.

#### Data on Care Seeking and Sources for Care

If women report that 1 or more of their children was ill in the last 2 weeks, they are asked if they sought treatment or advice for each child from any source. Care seeking is classified as any care sought outside the home. Because we focused on sources of care, this analysis focuses on care *outside* the home, not whether the child received an appropriate treatment (e.g., oral rehydration solution and zinc supplements, which could have been administered at home). Similarly, this analysis likely does not capture other medications kept and administered at home, whether or not they are appropriate.

If mothers report seeking treatment or advice outside the home, they are asked where they went. Their responses are classified into precoded categories that vary by country. To standardize categories across countries, we classified sources into public or private sector or “other” sources including traditional healers, friends, and family (Table). Public and private sector sources are further classified by whether the source was a health facility, a community health worker (public sector only), or a retail outlet (private sector only). We note that these categories, based on mother’s recall, may not perfectly capture precise sources, but we believe the categories used are broad enough to represent source groups with reasonable accuracy. We also note that these results reflect where sick child care was sought in the 2 weeks before the survey and may not reflect parents’ subsequent care-seeking destinations or where parents may prefer to seek care if barriers (geographic, financial, etc.) were removed.

**TABLE.** Source Categorizations Used in Care-Seeking Analysis of 24 USAID Maternal and Child Health Priority Countries

	**Public sector**	**Private sector**	**Other**
**Health facilities**	Hospitals Clinics Health posts	Private clinics, hospitals, and clinicians Nongovernmental and faith-based clinics	Traditional healers Friends or family members
**Community health workers and retail outlets**	Community health workers	Pharmacies Shops, markets	

Abbreviation: USAID, U.S. Agency for International Development.

#### Data on Equity

We examined results by socioeconomic status by using the DHS wealth quintiles, which divide the population surveyed in each country into evenly-sized quintiles based on their household assets.^12^ We used the bottom and top quintiles, respectively, to represent children and caregivers from the poorest 20% and wealthiest 20% of households in each country. We also examined results by urban and rural residence. We used the DHS classifications of urban and rural, which are based on the classifications used in each country.

### Analytic Methods

The unit of analysis for all research questions is children aged 0–59 months (under 5 years) old. All analyses used DHS survey sampling weights. To generalize results across countries, we multiplied the survey weights by a survey-specific constant to standardize the effective weighted sample size across countries. Thus, each country contributes equally to the regional and all-country averages, and results are not weighted more heavily toward surveys with larger sample sizes or populations. We considered weighting results by the population size of each country, but found that nearly three-quarters of the population-weighted sample would be from Asia because the Asian countries in our analysis are more populous, and noted that the 24 countries are not representative of any regions or larger geographies. There-fore, average estimates should be interpreted as averages across countries analyzed. Similarly, regional results are not representative of the entire region but should be interpreted as the average across countries analyzed in each region. All surveys are included in averages, but country-level results are suppressed if they are based on fewer than 50 unweighted cases. All analyses and visualizations were conducted in Stata version 14.2.

## RESULTS

Results presented here describe the prevalence of ARI symptoms, diarrhea, and/or fever among children 5 or younger and associated care-seeking levels and source patterns. In each section, we present regional averages for countries analyzed in Asia, East and Southern Africa, and West and Central Africa to summarize patterns observed in the data.

### Prevalence of Illness Classifications

Reports of ARI symptoms, diarrhea, and fever among children under 5 were common across the countries examined. The prevalence of these illness classifications ranged widely between countries (15.9%–46.9%). On average, 1 of 3 children (32.9%) experienced 1 or more of these 3 illness classifications in the 2 weeks before the survey.

**FIGURE 2.**
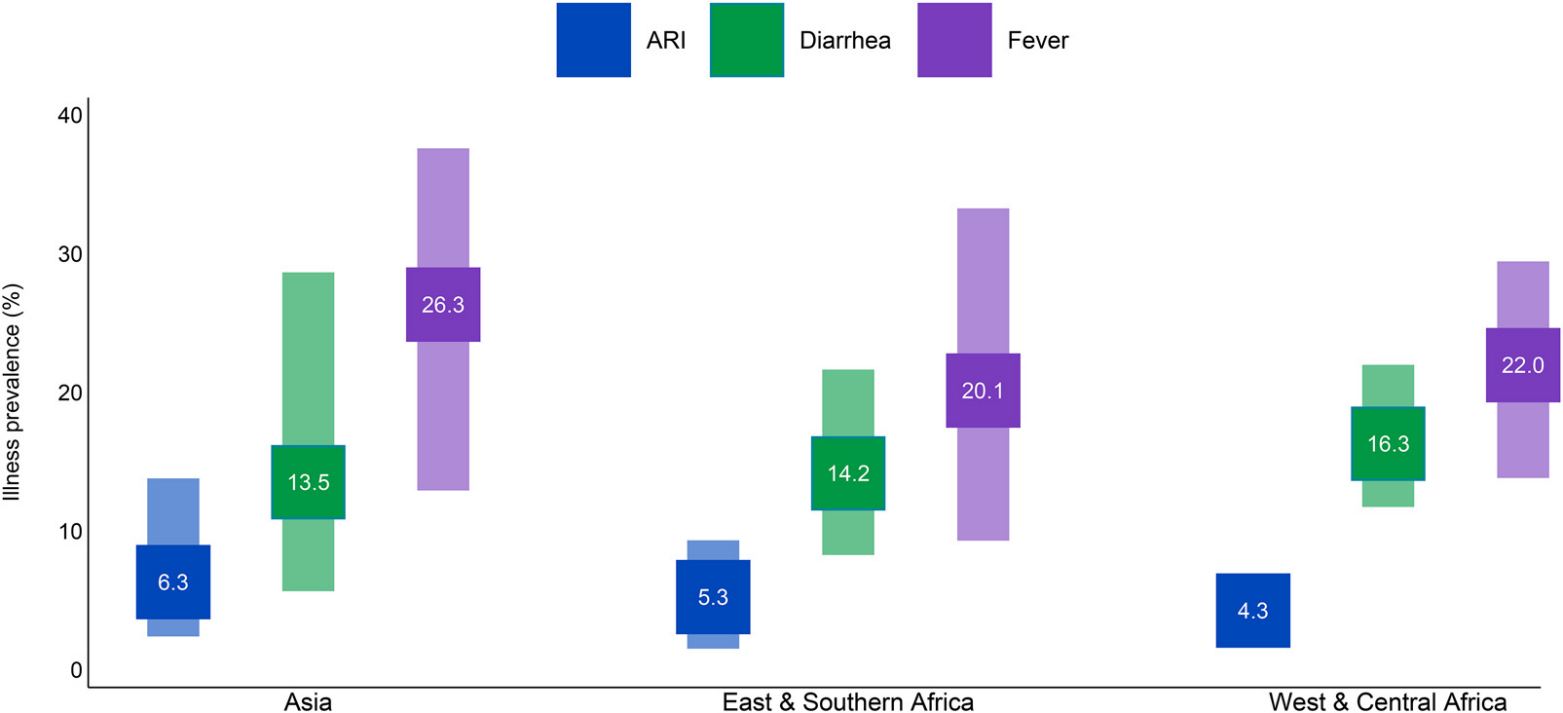
Prevalence of Acute Respiratory Infection Symptoms, Diarrhea, and Fever in USAID Maternal and Child Health Priority Countries, by Region^a^ Abbreviations: ARI, acute respiratory infection; USAID, U.S. Agency for International Development.^a^ The range of prevalence is indicated by the vertical bars. The mean is noted in each square.

Fever was the most common classification reported in all priority countries and regions. On average across priority countries, 23.4% of children under 5 experienced fever, 15.5% had diarrhea, and 5.8% experienced ARI symptoms (Figure 2).

Across all countries analyzed, the prevalence of fever (Figure 2) ranged widely: Madagascar had the lowest prevalence of fever (9.3%); both Bangladesh (36.8%) and Pakistan (37.6%) had the highest. Bangladesh was an outlier with a comparatively high prevalence of fever and for its low level (5.6%) of diarrhea. Yemen (31.2%) and Afghanistan (28.7%) stand out for diarrhea prevalence that was double the all-country average (15%). With the lowest levels on average, the range of ARI prevalence across countries was not as large; Nepal (2.4%), Mali (2.0%), and Mozambique (1.5%) were the countries with the lowest ARI prevalence, and Pakistan had the highest rate (13.8%).

### Prevalence of Comorbidities

Comorbidities—reports of multiple types of illness at the same time—were common among children in priority countries. Among the 3 classifications examined, nearly 1 in 5 (18.8%) children suffered from more than 1 symptom in the 2 weeks before the survey. Regionally, comorbidities were highest in the Asian countries analyzed (21.4%), compared to 15.9% in the sub-Saharan countries. Children in Haiti (34.9%) and Pakistan (34.1%) had the highest prevalence of comorbidity.

#### Equity Implications: Disparities in Illness Classification Prevalence

**FIGURE 3.**
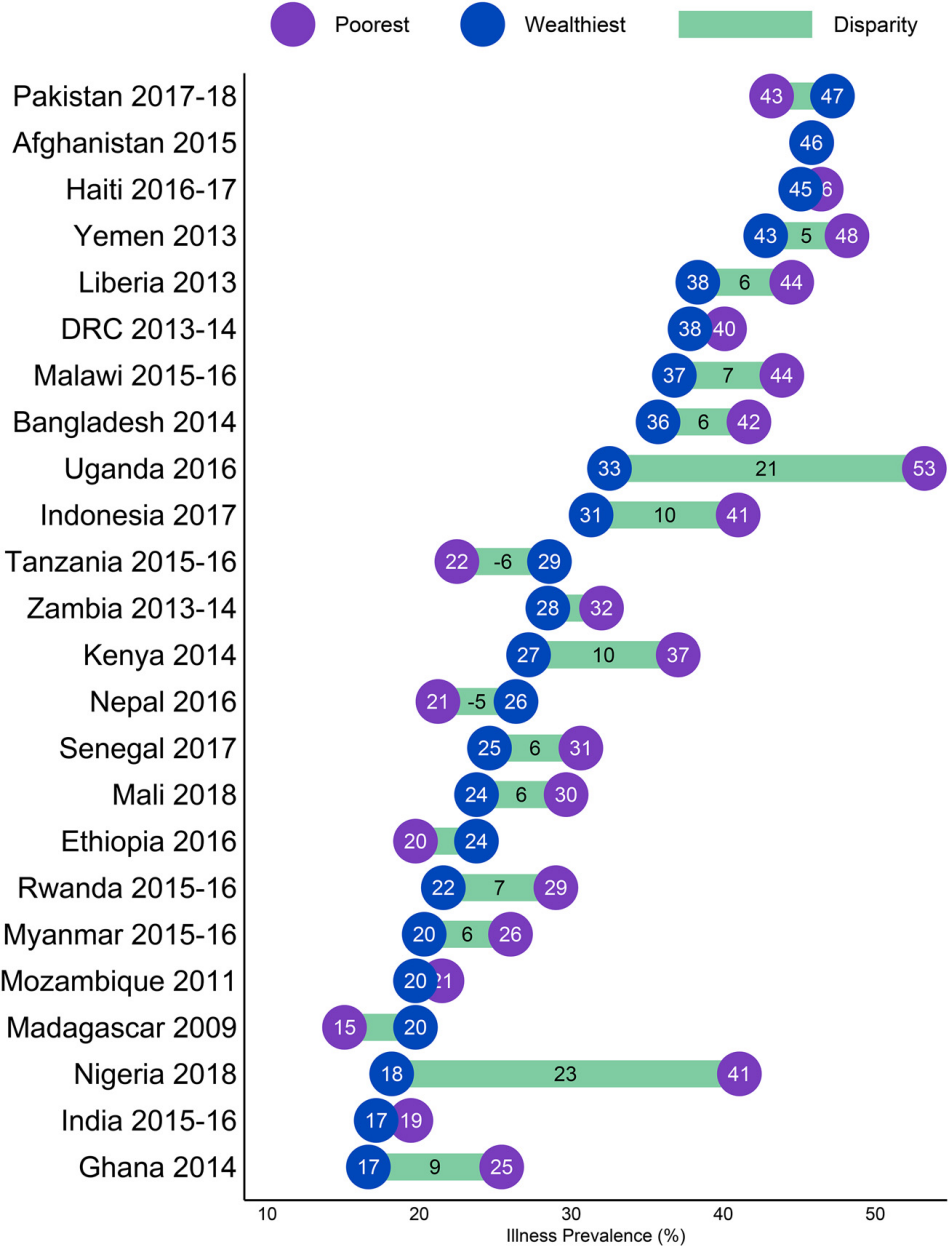
Prevalence of ARI Symptoms, Fever, and/or Diarrhea and Disparity Between Children in the Poorest and Wealthiest Households in USAID Maternal and Child Health Priority Countries^a^ Abbreviations: ARI, acute respiratory infection; DRC, Democratic Republic of the Congo; USAID, U.S. Agency for International Development.^a^ The bars depict the magnitude of the difference in reported prevalence between the poorest and wealthiest, with values shown when the magnitude is 5 or more percentage points.

Differences in illness classification prevalence between children living in urban and rural areas was small (less than 4 percentage points for all regions) but were more notable between children from the poorest and wealthiest households. On average, across all countries and all 3 illnesses classifications, prevalence was 4.5 percentage points higher among children in the poorest households (34.1%) than those in the wealthiest households (29.6%). By region, the disparity in prevalence of 1 or more illness classifications was largest in West and Central African countries (8.7 percentage points), followed by East and Southern Africa (3.9 points), and smallest in the countries analyzed in Asia (2.1 points), with substantial variation at the country level. The disparity for children in the poorest and wealthiest households in most countries was 10 percentage points or lower; the notable outliers were Nigeria and Uganda where the disparity between the wealthiest and poorest (22.9 and 20.7 percentage points, respectively) was more than double the disparity of any other country examined (Figure 3).

The magnitude of the disparity in illness classification prevalence between children from the poorest and wealthiest households differed somewhat by type of classification. The largest disparities were observed for fever: in West and Central African countries analyzed fever prevalence was 6.4 percentage points higher on average among the poorest households compared to the wealthiest households, and 4.8 percentage points higher among poorest than wealthiest households on average in the East and Southern African countries analyzed. Disparities in diarrhea prevalence were generally smaller than those for fever. The largest reported diarrhea prevalence disparities were observed in the West and Central African countries analyzed, particularly Nigeria and Senegal (12.5 and 9 percentage points, respectively). Outside of this region the average disparity for reported diarrhea prevalence was 1.4 percentage points in the East and Central African countries analyzed and was 1.2 percentage points in the Asian countries analyzed. The smallest disparities between poorest and wealthiest children observed in priority countries were for reported ARI symptoms: 1.7 percentage points on average across all countries analyzed, with West and Central African countries having the smallest average disparity (0.9 percentage points). For more details, see Supplemental Figures.

### Out-of-Home Care Seeking

**FIGURE 4.**
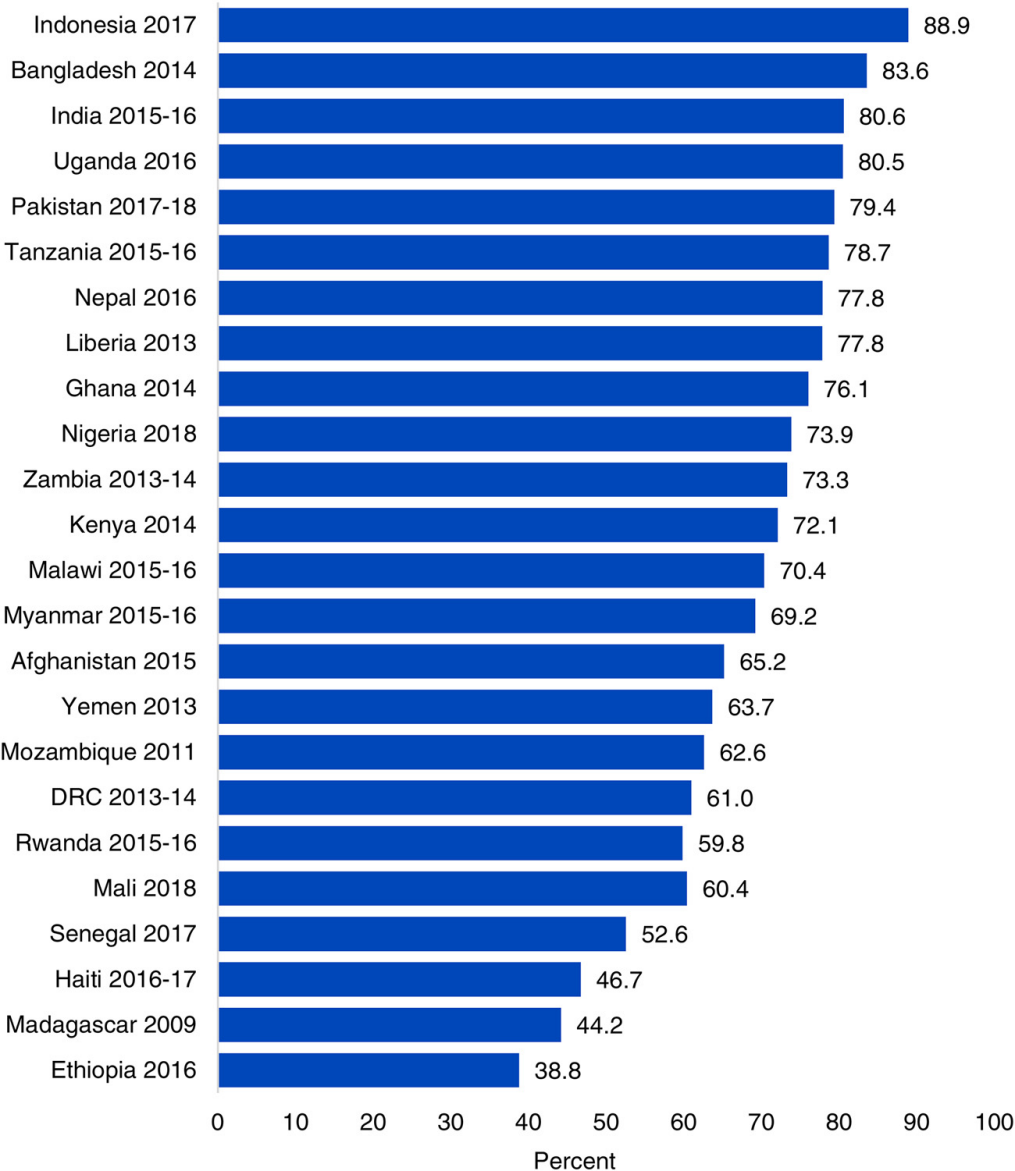
Out-of-Home Care-Seeking Levels by Country Among Caregivers of Sick Children in USAID Maternal and Child Health Priority Countries Abbreviations: DRC, Democratic Republic of Congo; USAID, U.S. Agency for International Development.

On average across all USAID priority countries, most caregivers (68.2%) sought treatment outside of the home when their children experienced 1 or more of the 3 classifications examined in this analysis. Overall, care seeking was highest in Asia (77.8%) with lower levels across Africa (66.9% in West and Central Africa, 64.5% in East and Southern Africa). At the country level, care-seeking levels varied widely (Figure 4) with the lowest levels of care seeking in Ethiopia (38.8%) and Madagascar (44.2%) and the highest levels of care seeking in Indonesia (88.9%) and Bangladesh (83.6%).

#### Care Seeking by Illness Classification Type

Although care-seeking levels vary substantially across countries, they are similar across illness classifications. Care-seeking levels were highest for ARI (70.4%) and fever (68.5%) and slightly lower for diarrhea at 63.3%. This pattern, which was also observed at the regional level, may be partially driven by the fact that diarrhea can be effectively managed at home with oral rehydration solutions and zinc supplements.

#### Equity Implications: Gaps in Care Seeking by Urbanicity and Wealth

**FIGURE 5.**
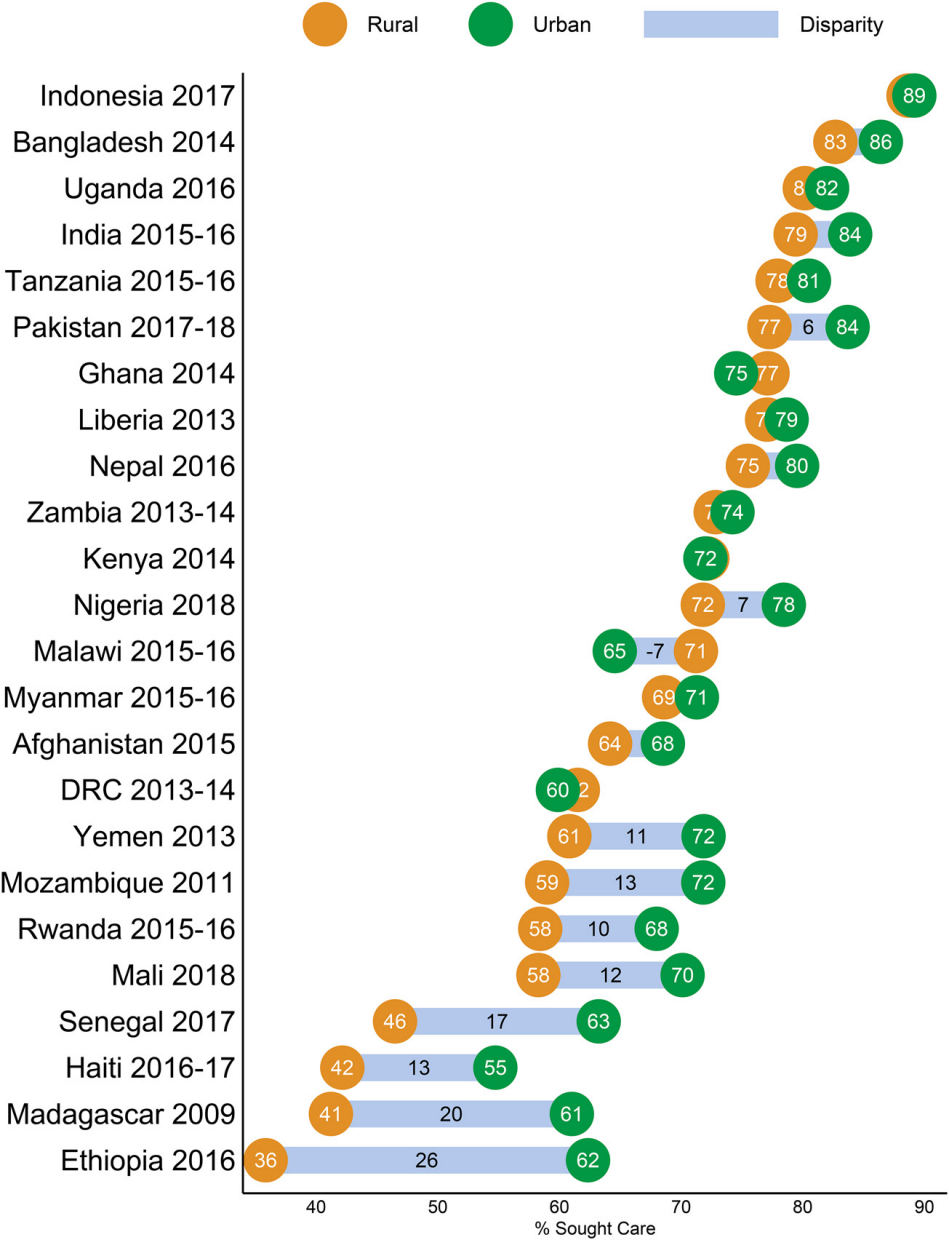
Differences in Care Seeking Levels Among Caregivers from Urban and Rural Households in USAID Maternal and Child Health Priority Countries^a^ Abbreviations: DRC, Democratic Republic of the Congo; USAID, U.S. Agency for International Development.^a^ The bars depict the magnitude of the difference in urban and rural care seeking, with values shown in cases where the magnitude is 5 or more percentage points.

Across all countries analyzed there were equity-related gaps in care seeking. The care-seeking level in rural areas was 6.3 percentage points lower on average than the urban care-seeking level, with a high-degree of variability across countries (Figure 5). In general, differences were smallest where overall care-seeking levels were highest. For example, in Indonesia, 89% of both urban and rural caregivers sought sick child care outside the home. The largest urban-rural care-seeking gaps were in countries with lower overall levels of care seeking, particularly in Madagascar where care was sought for 41.2% of rural children and 61% of urban children—a gap of 19.8 percentage points—and in Ethiopia, which had the lowest overall levels of care seeking and the greatest urban-rural disparity, at 26.5 percentage points.

**FIGURE 6.**
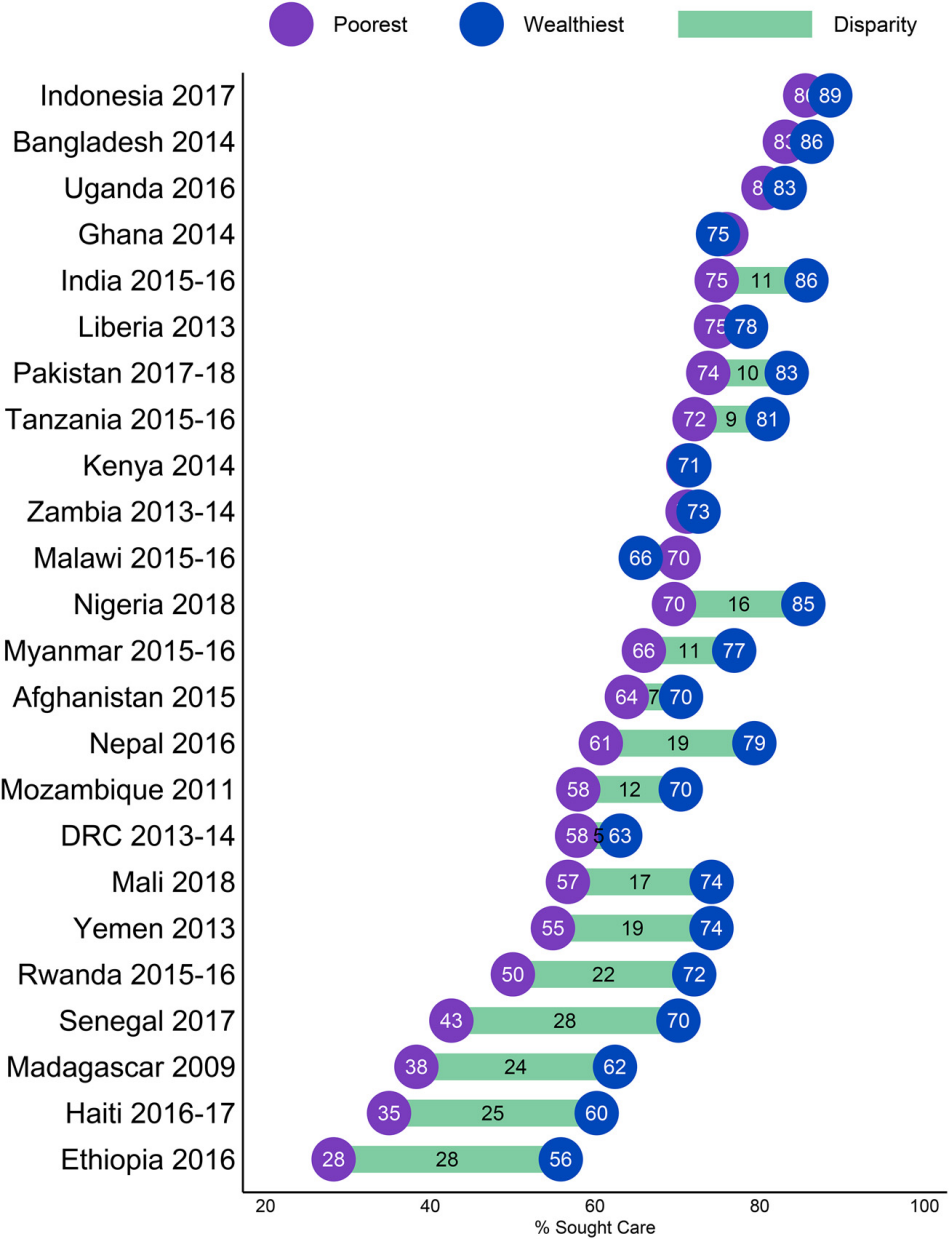
Differences in Care Seeking Among Caregivers With Sick Children From the Poorest and Wealthiest Households in USAID Maternal and Child Health Priority Countries, by Country^a^ ^a^ The bars depict the magnitude of the difference between the poorest and wealthiest, with values shown in cases where the magnitude is 5 or more percentage points.

There were also substantial disparities in care seeking for children from the poorest and wealthiest households (Figure 6). Across all countries, the average care-seeking level for any illness classification was 11.3 percentage points higher for caregivers from the wealthiest households (74.4%) than from the poorest households (63.1%). Among the Asian countries analyzed, the disparity in care seeking ranged from 3.0 to 11.0 percentage points. Sub-Saharan African countries, on the other hand, had a much wider range in care-seeking gaps. In some cases (Zambia, Kenya, Ghana, and Malawi) care seeking among the poorest caregivers was similar to or higher than care seeking among the wealthiest caregivers, but in other cases (Rwanda, Madagascar, Senegal, Ethiopia) the wealth gap in care seeking exceeded 22 percentage points or double the size of the gap for priority countries overall.

### Sources of Care

On average, across countries analyzed, half of caregivers (51.1%) who sought out-of-home care for their children’s most recent illness reported that they went to a public source; 42.5% reported that they went to a private sector source, 4.9% reported that they sought care from “other” sources (including informal providers, traditional healers, friends, or family members), and 1.5% reported that they sought care from both public and private sources. This care-sourcing pattern was very consistent across classifications; for example, the proportion of caregivers who used private sector sources was 41.2% when their child had diarrhea, 43.6% for fever, and 42.2% for ARI symptoms. This consistency in both levels of care seeking and treatment sources by illness classification may indicate that the symptoms children were experiencing did not factor heavily in caregivers’ decision making about where to seek out-of-home advice and treatment. Because sources did not vary by classification, the remaining results in this section focus on sources used for children sick with any of the 3 illnesses classifications.

**FIGURE 7.**
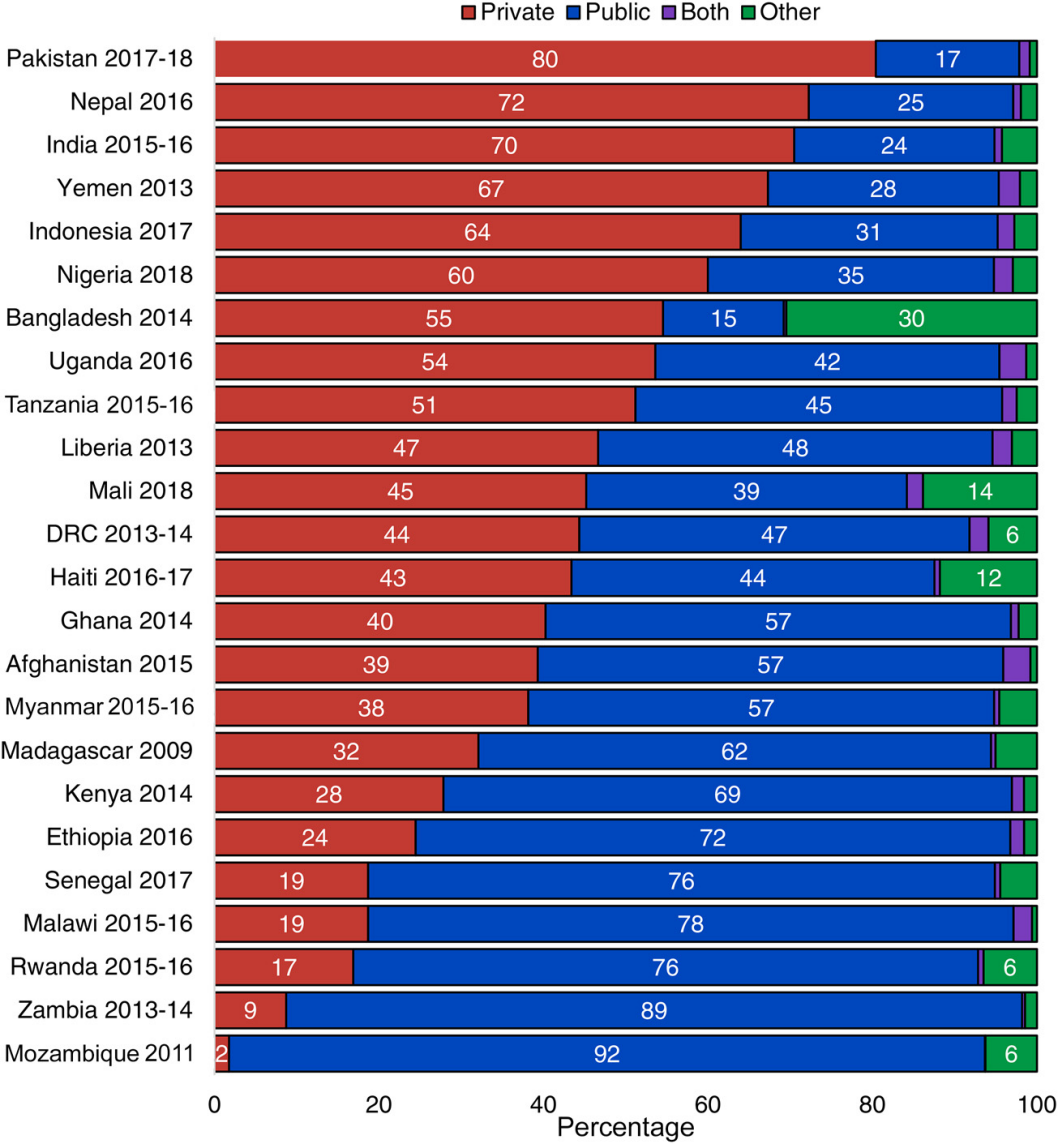
Sources for Sick Child Care in USAID Maternal and Child Health Priority Countries, by Country Abbreviations: DRC, Democratic Republic of the Congo; USAID, U.S. Agency for International Development.

However, sources of sick child care did vary within and across regions (Figure 7). In Asian countries analyzed, the private sector was the dominant source of care, with 59.9% of caregivers seeking care from this source. Pakistan had the highest level of private sector care seeking (80.4%) of any country analyzed, and Myanmar and Afghanistan’s private sector care-seeking levels (39.3% and 38.2%, respectively) were substantially lower than other countries in that region. By contrast, in East and Southern Africa, the public sector was the source of care for a large majority (69.6%) of sick children, and in Mozambique, the public sector was the source of care for almost all (91.9%) children. In West and Central Africa, there was a more even split in care seeking from public (52.1%) and private (42.5%) sources on average. There was much variation at the country level, for example, in Nigeria where the private sector was the source of out-of-home care for the majority (60%) of caregivers. Although relatively few caregivers sought care from other informal sources, there were 2 notable exceptions: in Bangladesh, the level of care sought from other sources (predominantly “unqualified doctors”) was 30.5%, and in Mali it was 13.8% (predominantly “traditional practitioners”).

### Equity Implications: Comparing Sources of Care by Urbanicity and Wealth

**FIGURE 8.**
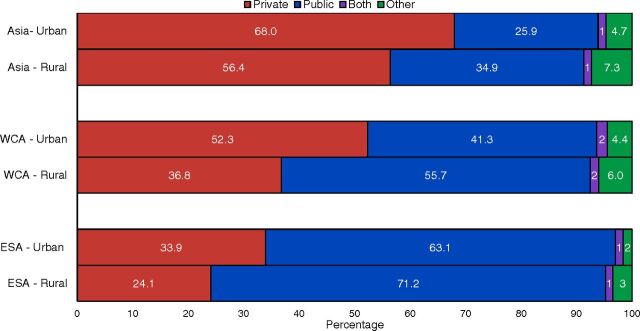
Sources for Sick Child Care in USAID Maternal and Child Health Priority Countries, by Region and Urbanicity Abbreviations: DRC, Democratic Republic of the Congo; ESA, East and Southern Africa; USAID, U.S. Agency for International Development; WCA, West and Central Africa.

Analysis of reported sources for sick child care across all countries and regions showed similar patterns when disaggregated by urban versus rural residence (Figure 8). In each region, caregivers in rural areas had lower levels of care seeking from private sources and higher levels of care seeking from public and informal sources.

The patterns of sources of care used by caregivers from the poorest and wealthiest quintiles was nearly identical to sources used by rural and urban caregivers: a consistent pattern of higher private sector use among the wealthiest and higher public and informal sector use among the poorest caregivers in each region. Despite this overall pattern, analysis across countries revealed that caregivers of all wealth levels—even the poorest caregivers—sought care for their children from private sector sources. On average across priority countries, 35.4% of caregivers from the poorest households in each country sought care from private sector sources and 55.1% of caregivers from the wealthiest households.

### Types of Public and Private Sector Sources

The public and private sectors are not homogenous and are made up of different types of health providers. In the public sector, sources included health facilities (hospitals, clinics, health posts) and community health workers (CHWs). In the private sector, reported sources included health facilities (hospitals, doctors, and private for-profit, nongovernmental organizations, and faith-based clinics) and private retail outlets (pharmacies, shops, and markets). When seeking care from a health facility, caregivers are likely to interact with clinically trained health professionals. In contrast, prior research has shown that retail outlets may be less likely to have adequately trained health professionals, and providers may have limited access to or training on current treatment and counseling policies and guidelines, which could potentially result in a substandard quality of medical care.^13^^–^^15^

Among caregivers who recently sought care from public sector sources, nearly all (95.6%) reported that they received care at a health facility rather than from a CHW. Fewer than 5% of caregivers in most countries reported that they sought help from a CHW. However, there were 2 notable outliers; in Indonesia and Rwanda, 26.7% and 26.3% of caregivers, respectively, sought care or advice from a CHW.

Overall, care seekers who used the private sector consulted with providers in health facilities (48.8%) and retail outlets (52.4%) at nearly equal levels. Although this split did not vary by illness classification, there was somewhat more variation at the regional level. In the Asian and East and Southern African countries analyzed, a majority of caregivers who reported private-sector care sourcing went to a health facility (61.4% and 55.7%, respectively). By contrast in the West and Central African countries analyzed, a minority of caregivers (22.1%) reporting private sector care seeking went to a health facility.

### Equity Implications: Comparing Private Sources of Care by Urbanicity and Wealth

**FIGURE 9.**
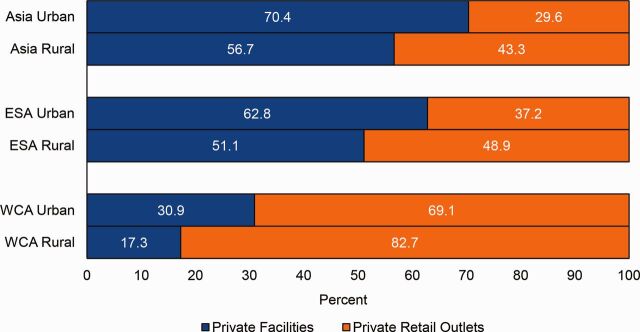
Types of Private Providers Consulted in Urban and Rural Areas, by region Abbreviations: DRC, Democratic Republic of the Congo; ESA, East and Southern Africa; USAID, U.S. Agency for International Development; WCA, West and Central Africa.

Among caregivers who sought care from private sector sources, the types of providers seen varies by urban/rural residence and socioeconomic status. This section examines the use of health facilities versus pharmacies and retail outlets among caregivers who used private sector sources. By urban and rural residence, health facility use versus retail outlet use followed a similar pattern across all regions (Figure 9). In each region, caregivers residing in rural areas had lower levels of care seeking from private health facilities and higher levels of care from private retail outlets. This pattern may reflect a distribution of health facilities that favors urban areas.

**FIGURE 10.**
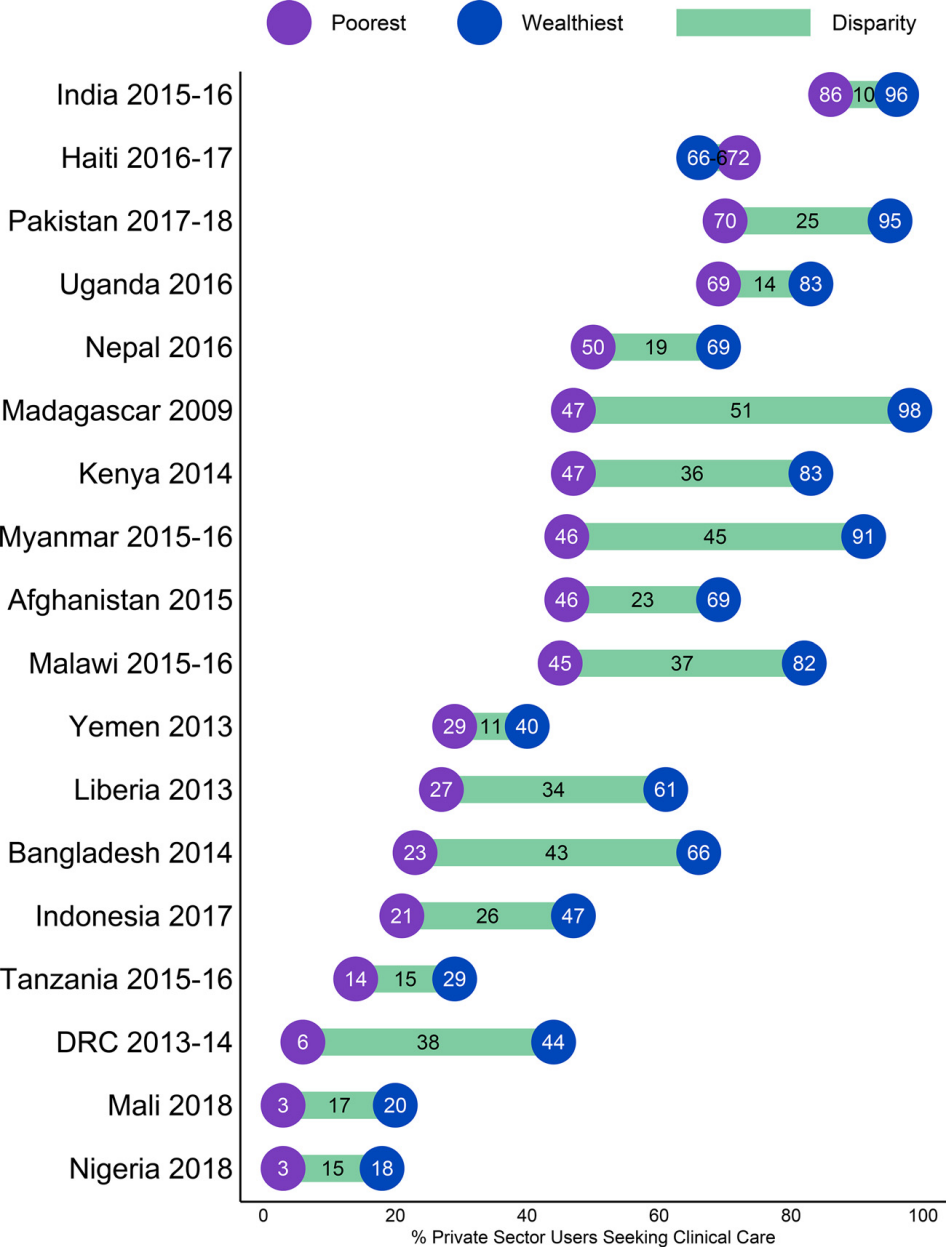
Levels of Private Sector Health Facility Care Seeking Among the Poorest and Wealthiest Caregivers Who Sought Care from Private Sector Sources in USAID Maternal and Child Health Priority Countries, by Country^a^ Abbreviations: DRC, Democratic Republic of the Congo; USAID, U.S. Agency for International Development.^a^ The bars depict the magnitude of the difference between the poorest and wealthiest households.

Similarly, we found that within the 35.4% of the poorest caregivers and 55.1% of the wealthiest caregivers who sought care from the private sector, the poorest and wealthiest used different sources within the sector. On average across countries, 62.4% of the wealthiest caregivers who reported private sector care seeking went to a health facility. The data reflect a converse scenario for those with the lowest socioeconomic status; among this segment a majority (57.2%) of the poorest private sector care seekers used retail outlets. On average, the gap in private health facility care seeking between the wealthiest and poorest caregivers was 33.5 percentage points in the Asian countries analyzed and 31 percentage points in the West and Central African countries analyzed. The average gap in private health facility care seeking in the East and Southern African countries analyzed was smaller (17.9 percentage points). Except for Haiti, this overall pattern holds for all priority countries (Figure 10). Disparities were particularly large in Madagascar and Bangladesh where the gap in private health facility care seeking between the wealthiest and the poorest households was 51.4 and 42.2 percentage points, respectively.

## DISCUSSION

Reports of treatable and preventable illness symptoms were extremely common across USAID’s maternal and child priority countries, affecting 1 in every 3 children under age 5. Given this high illness burden, stewards of the public and private sectors—including nongovernmental organizations, civil society, donors, and implementing partners—must come together to understand sources of sick child care and work to improve access to and quality of affordable care.

Although the majority of caregivers (68.2% on average) sought treatment or advice when their children were sick, there are still many areas where progress is needed, especially to improve equity. For example, in nearly half of the priority countries, more than one-third of the poorest caregivers did not seek care outside the home for their sick children. On average across countries, there was a 6.3 percentage point disparity in care-seeking levels comparing rural with urban families and an 11.3 percentage point disparity between the wealthiest versus poorest families. Potential barriers to seeking timely advice and treatment may include poor availability, limited access, un-affordability, and inadequate information about illness danger signs. In countries with overall low care seeking or low care seeking among specific population groups, additional exploration into these potential barriers is warranted.

Both public and private sectors were critically important for families seeking care, reinforcing the need for sectors to complement each other to provide equitable access to high-quality care.

Among those families that did seek care, both the public (51.1%) and private (42.5%) sectors were critically important, reinforcing the need for health actors across sectors to complement one another and ensure that there is equitable access to high-quality care for all population segments. While the majority of the poorest (56.6%) and rural (52%) care seekers accessed advice and treatment from public sources, more than 1 in 3 of the poorest (35.4%) and rural (37%) care seekers used private health facilities, pharmacies, or shops. Poorer and rural families may rely on private sources dues to increased convenience, quality perception, or even affordability, for example if subsidized products are available in the private nonprofit sector.

Although use of private retail outlets like pharmacies and shops was quite high (52.3%) overall among care seekers who used the private sector, the poorest care seekers in this group sought much more care (59.2%) from retail outlets than wealthy care seekers who used the private sector (37.4%). This is an important disparity because these types of retail outlets may be less likely to be staffed by providers trained with up-to-date guidelines, suggesting that the poorest care seekers may be at risk of obtaining poorer quality care than their wealthier counterparts. As efforts expand in the private sector to strengthen the quality of integrated management of sick child care, this finding is key to ensure that these efforts reach both private facilities as well as private pharmacies, shops, and other retail outlets.

Among care seekers who sought care in the public sector, the large majority (95.6%) consulted health facilities rather than CHWs. This aligns with results from a Hodgins et al. analysis (2013) in which they concluded that it^10^:

*may be inappropriate to focus program efforts on community health workers to the exclusion of more widely used sources of care*.

When sharing our analysis results with stakeholders in a selection of priority countries, stakeholders were particularly surprised to see low use of CHWs given the amount of resources dedicated to community case management. A potential limitation pertaining to reporting of CHWs is that DHS asks women where they “go,” so respondents may not report a CHW if they saw a CHW at a health post or if they did not physically “go” outside of their home for care. In addition, it is possible that CHWs provide advice but do not consistently stock appropriate treatment, so caregivers may go where they know supplies can be readily obtained. Before refocusing program efforts away from CHW case management programs, we suggest conducting country-specific explorations into CHW programs to determine if and how they are used and barriers to successful operation.

### Using the Data

It is critical to examine childhood illness classifications and care-seeking patterns at the country level where policy makers, advocates, and civil society are charged with implementing cost-effective and sustainable policies and programs that will lower childhood mortality and meet Sustainable Development Goals. These data are pertinent for government actors employing strategies to efficiently mobilize domestic resources across sectors. Interpreting findings at the country level requires a robust understanding of national and subnational factors including health financing, health system functioning, governance and policy, sociocultural norms, and ongoing campaigns or interventions. With this contextual knowledge, stakeholders can use these data to better understand and begin to tackle challenges such as low care-seeking levels, inequities in care seeking, and poor access to particular sources of care.

To assist country stakeholders in interpreting and using these findings, we have presented data through accessible infographics and data visualizations. All of our data are housed in an online interactive data visualization tool—called PrivateSectorCounts.org—that allows users to explore findings in their own country or across countries and make comparisons across demographic characteristics. Further, we have disseminated findings through country-specific briefs and annotated PowerPoint presentations, available on SHOPSPlusProject.org.

The authors worked with several USAID Missions to interpret findings together based on their nuanced country knowledge and subsequently disseminated analysis findings together via 3 regional webinars. This process yielded fruitful insights and programmatic recommendations, exemplified through the quotes below:

*One key thing … is advocating for expanding health financing options to deliver services. These results show that the private sector has a big role in delivering services, and the government needs to put in more effort to ensure that they strengthen the private [sector] equally to the public sector. … These results give us a powerful tool to advocate for a comprehensive health financing strategy.* —USAID/Uganda Representative

*In India, it's surprising that even 70% of the poorest seek care from the private sector … Our understanding was that amongst the poor, the public sector was dominant. But, this data shows us that poor also get care from the private sector. This gives us a clue about improving the care in the private sector.* —USAID/India Representative

### Limitations

DHS data are self-reported, so it is possible that there are errors or misclassifications in sources and levels of care used. For example, it is possible that respondents do not consider interactions such as buying medication in a shop to be “advice or treatment outside the home,” which could lead to underreporting of care-seeking levels, though given the high levels of reported care seeking from pharmacies and retail outlets, we anticipate any underreporting would likely be minimal. In addition, respondents are asked where they “go” for care, not whom they see, so if respondents see a CHW at a health post, for example, the response may be recorded as health post (categorized as a type of health facility) rather than as a CHW. The low levels of reported CHW care provision may also be related to the fact that the DHS only includes a standard response category for public sector CHWs, rather than response categories both for public and private sector CHWs. Local understandings of who is a CHW (some of whom might be reported by respondents as "traveling nurses" instead, for example) may further complicate efforts to understand care-seeking levels through CHWs via this analysis alone. We also note that data were collected from mothers, though a different caregiver may have been the one to seek care—this is especially likely in countries where women often have restricted mobility as in Afghanistan. If a different caregiver sought care for the sick child, the mother may not have had complete information on care-seeking sources.

Additionally, this analysis does not include data about preferences for sources of care. The sources reported are those used and may not be the preferred source of care if all sources were available and accessible to the respondent. As such, conclusions cannot be drawn about preferred care sources from these data.

## CONCLUSION

The public and private sectors both play key roles in treating sick children. Stakeholders across sectors must collaborate and strategize to reach all population segments with high quality child health services and work toward reducing disparities in care-seeking behaviors. Given the high use of private retail outlets—namely pharmacies, drug shops, and markets—efforts to ensure knowledge of and adherence to appropriate integrated management of childhood illness protocols in these outlets should continue and be strengthened, including through additional research when warranted. Cross-sectoral communication and joint problem solving is particularly critical in the context of the COVID-19 pandemic and other external shocks that create health system and health care-seeking constraints. Such cross-sectoral efforts will build clinical and institutional capacity and more efficiently allocate resources, ultimately resulting in stronger, more resilient health systems.

## Supplementary Material

20-00115-Bradley-Supplemental-Figures.pdf

## Notes

**Peer Reviewed**

**First published online:** September 4, 2020

## References

[1] United Nations Inter-agency Group for Child Mortality Estimation. *Levels & Trends in Child Mortality: Report 2019*. United Nations Children’s Fund; 2019. Accessed August 4, 2020. https://www.unicef.org/sites/default/files/2019-10/UN-IGME-child-mortality-report-2019.pdf

[2] Chao F, You D, Pedersen J, Hug L, Alkema L. National and regional under-5 mortality rate by economic status for low-income and middle-income countries: a systematic assessment. Lancet Glob Health. 2018;6(5):e535–e547. 10.1016/S2214-109X(18)30059-7. 29653627 PMC5905403

[3] U.S. Agency for International Development (USAID). *USAID POLICY FRAMEWORK: Ending the Need for Foreign Assistance*. USAID; 2019. Accessed August 4, 2020. https://www.usaid.gov/policyframework/documents/1870/usaid-policy-framework

[4] U.S. Agency for International Development (USAID). *Acting on the Call: A Focus on the Journey to Self-Reliance for Preventing Child and Maternal Deaths*. USAID; 2019. Accessed August 4, 2020. https://www.usaid.gov/sites/default/files/documents/1864/USAID_2019_AOTC.pdf

[5] Department for International Development (DFID). *Department for International Development Annual Report and Accounts 2018–19*. DFID; 2019. Accessed August 4, 2020. https://assets.publishing.service.gov.uk/government/uploads/system/uploads/attachment_data/file/815787/ARA-2019.pdf

[6] The Gates Foundation. Maternal, Newborn and Child Health: Strategy Overview. Accessed August 4, 2020. https://www.gatesfoundation.org/What-We-Do/Global-Development/Maternal-Newborn-and-Child-Health

[7] Global Financing Facility (GFF). *Private Sector Engagement*. GFF; 2016. Accessed August 4, 2020. https://www.globalfinancingfacility.org/sites/gff_new/files/documents/Private%20Sector%20Engagement%20Strategy.pdf

[8] U.S. Agency for International Development (USAID). Private-Sector Engagement Policy. Last updated April 1, 2019. Accessed August 4, 2020. https://www.usaid.gov/work-usaid/private-sector-engagement/policy

[9] Montagu D, Visconti A. Health care utilization around the world. Presentation presented at: International Health Economics Association Pre-Congress Symposium; July 9, 2011; Toronto, ON, Canada. Accessed August 4, 2020. https://www.shopsplusproject.org/resource-center/health-care-utilization-around-world

[10] Hodgins S, Pullum T, Dougherty L. Understanding where parents take their sick children and why it matters: a multi-country analysis. Glob Health Sci Pract. 2013;1(3):328–356. 10.9745/GHSP-D-13-00023. 25276548 PMC4168586

[11] Winter R, Wang W, Florey L, Pullum T. *Levels and Trends in Care Seeking for Childhood Illness in USAID MCH Priority Countries*. ICF International; 2015. Accessed August 4, 2020. https://dhsprogram.com/pubs/pdf/CR38/CR38.pdf

[12] Rutstein SO, Johnson K. *The DHS Wealth Index. DHS Comparative Reports No. 6*. ORC Macro; 2004. Accessed August 4, 2020. https://dhsprogram.com/pubs/pdf/CR6/CR6.pdf

[13] Wafula FN, Miriti EM, Goodman CA. Examining characteristics, knowledge and regulatory practices of specialized drug shops in Sub-Saharan Africa: a systematic review of the literature. BMC Health Serv Res. 2012;12(1):223. 10.1186/1472-6963-12-223. 22838649 PMC3520114

[14] Smith F. The quality of private pharmacy services in low and middle-income countries: a systematic review. Pharm World Sci. 2009;31(3):351–361. 10.1007/s11096-009-9294-z. 19343530

[15] Miller R, Goodman C. Do chain pharmacies perform better than independent pharmacies? Evidence from a standardised patient study of the management of childhood diarrhoea and suspected tuberculosis in urban India. BMJ Glob Health. 2017;2(3):e000457. 10.1136/bmjgh-2017-000457. 29018588 PMC5623271

