# Supplementary figures and images for: Where Do Caregivers Take Their Sick Children for Care? An Analysis of Care Seeking and Equity in 24 USAID Priority Countries
Source: Glob Health Sci Pract. 2020 Sep 30;8(3):518–33. doi: 10.9745/GHSP-D-20-00115 (PMC7541105; doi:10.9745/GHSP-D-20-00115)

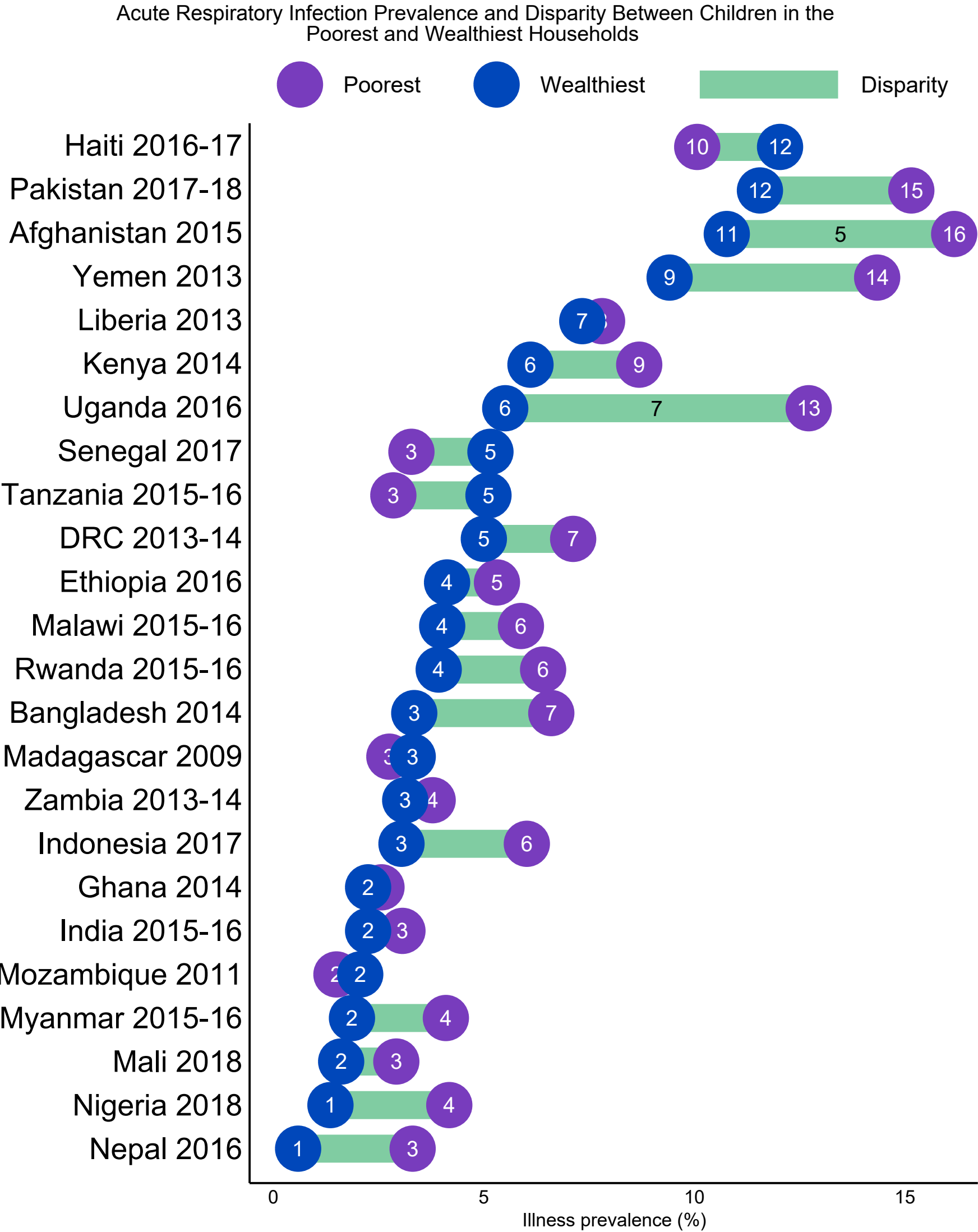

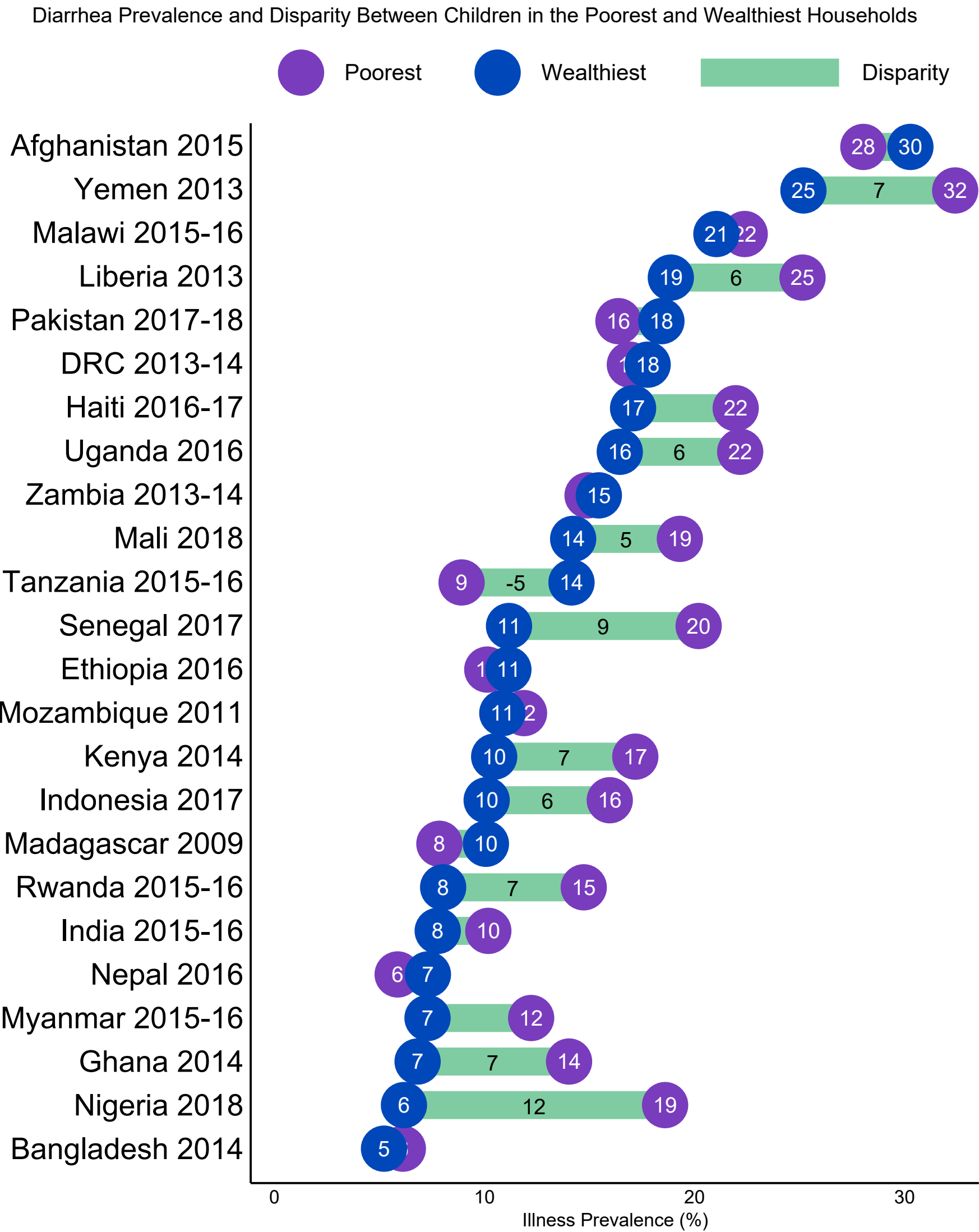

Fever Prevalence and Disparity Between Children in the Poorest and Wealthiest Households

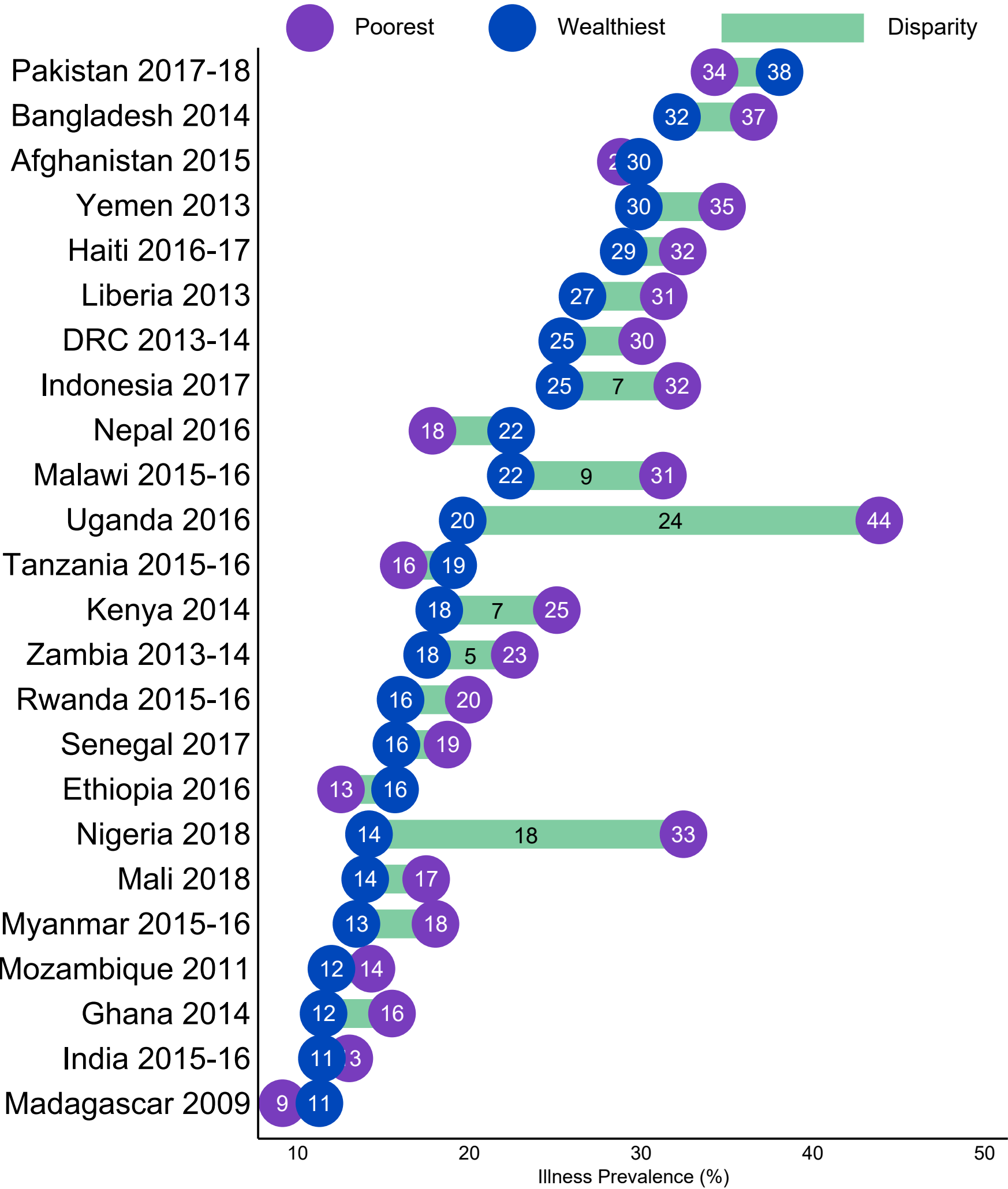

Supplement: 20-00115-Bradley-Supplemental-Figures.pdf [file 20-00115-Bradley-Supplemental-Figures.pdf]
